# Characterization of Protein Expression and Signaling Pathway Activation That May Contribute to Differential Biological Functions in Porcine Arterial and Venous Smooth Muscle Cells

**DOI:** 10.3390/ijms26073110

**Published:** 2025-03-28

**Authors:** Eyla C. Arteaga, Christine Wai, Unimunkh Uriyanghai, Medha Rudraraju, Huanjuan Su, Vinay A. Sudarsanam, Samuel O’Brien Haddad, John S. Poulton, Prabir Roy-Chaudhury, Gang Xi

**Affiliations:** 1 UNC Kidney Center, Division of Nephrology and Hypertension, Department of Medicine, The University of North Carolina at Chapel Hill, Chapel Hill, NC 27599, USA; eylae@med.unc.edu (E.C.A.); cwai@email.unc.edu (C.W.); unimunckh_uriyanghai@med.unc.edu (U.U.); huanjuan_su@med.unc.edu (H.S.); vinaysud@email.unc.edu (V.A.S.); samuel_haddad@med.unc.edu (S.O.H.); poultonj@email.unc.edu (J.S.P.); prabir_roy-chaudhury@med.unc.edu (P.R.-C.); 2 Department of Biology, Drexel University, Philadelphia, PA 19104, USA; mr3745@drexel.edu

**Keywords:** vascular smooth muscle cells (VSMCs), vein, artery, arteriovenous fistulae (AVF), stenosis, dedifferentiation

## Abstract

Arteriovenous fistulae (AVF) are the preferred mode of dialysis vascular access but have a maturation failure rate of over 50%. Venous segment stenosis is one of the main reasons. However, the reasons for the prevalence of stenosis in the venous segment, as opposed to the arterial segment, remain unknown. We hypothesize that differences in the protein expression profiles and in the activation of key signaling pathways of the vascular smooth muscle cells (VSMCs) in these two segments may contribute to this difference. In this study, arterial porcine VSMCs (ApSMCs) and venous porcine VSMCs (VpSMCs) were isolated to examine relevant protein expression and cell signaling pathway activation via Western blots and immunofluorescent staining. Our results revealed that growth-medium-stimulated cell proliferation was significantly higher in VpSMCs compared to ApSMCs, but no difference was detected in PDGF-BB-stimulated proliferation. VpSMCs migration was significantly greater than ApSMCs under both the serum-free condition and in the growth medium condition. In addition, VpSMCs appeared more susceptible to PDGF-BB-stimulated cell dedifferentiation. Furthermore, P53, vitronectin, collagen 1A1 and integrin β3 had lower expression in VpSMCs, whereas fibronectin, FAK, VCAM-1, MMP-9, TIMP-2 and TIMP-3 had higher expression as compared to ApSMCs. Meanwhile, PDGF-BB stimulated P38, JNKs and AKT activation, which were more potent in VpSMCs. Our results identify significant differences between VpSMCs and ApSMCs in the expression of functional proteins and the activation of signaling pathways, which may be responsible for more aggressive venous—as compared to arterial—stenosis in the clinical setting of arteriovenous access or other similar procedures.

## 1. Introduction

Cell plasticity is an important feature of vascular smooth muscle cells (VSMCs) [1] that results in adverse vessel remodeling after injury or in the case of vascular disease due to malfunctions of VSMCs. Smooth muscle cells (SMCs) execute different functions in different organs, such as those in the vascular, gastrointestinal and respiratory systems. A recent single cell transcriptomic study demonstrated the high-level differences in VSMCs from different organs and, importantly, the subtle differences in VSMCs within the same organ due to exposure to different environmental cues [2]. One example of this is the difference between arterial VSMCs and venous VSMCs. In the clinical setting of coronary artery bypass grafting, it has been well accepted that the use of the left internal mammary artery to bypass the left anterior descending artery is considered the “gold standard” of coronary artery revascularization based on outcomes [3]. Meanwhile, arteriovenous fistulae (AVF) have been the preferred method for dialysis treatment but have a failure rate of over 50% during maturation. One of the main reasons for this failure is stenosis in the venous segment, which is characterized by VSMCs’ phenotypic switch from a contractile, differentiated state to a synthetic, dedifferentiated state that results in neointimal hyperplasia.

Several previous studies have tried to investigate the differences between venous and arterial VSMCs. Using VSMCs from patient explants of a paired saphenous vein (SV) and internal mammary artery (IMA), a study demonstrated high proliferation of venous VSMCs in response to FCS, PDGF and bFGF, and high basal pERK1/2 activation in SV-VSMCs. However, no differences in the migration rate, matrix metalloproteinase (MMP)-2 secretion or MMP-9 secretion were detected in these two subtypes of cells [4]. In contrast, a study using VSMCs isolated from the jugular veins and carotid arteries of male white New Zealand rabbits found that venous VSMCs demonstrated enhanced proliferation, migration and collagen synthesis but decreased adhesion to collagen and fibronectin compared to arterial VSMCs. Higher levels of MMP-2, MMP-9 and tissue inhibitor of metalloproteinase-1 and -2 (TIMP-1 and -2) were detected in venous VSMCs [5]. Although these studies reveal some differences between these two subtypes of VMSCs, because of the discrepancies between studies, we still lack a clear understanding of the key differences between these cells.

Since a growing body of evidence supports that pigs are a good animal model to study human pathophysiology [6,7], we chose to address this knowledge gap by isolating VSMCs from porcine jugular veins and carotid arterials. A detailed comparison of venous and arterial porcine VSMCs was performed, including cell proliferation, cell migration and phenotypic switching of these two subtypes of cells under different conditions. To explore the potential underlying mechanisms that are responsible for their different biological functions, we determined the expression of many proteins relevant to their primary functions, including cell adhesion proteins, cell cycle related proteins, extracellular matrix (ECM) proteins, integrin proteins, MMPs and TIMPs. We also examined the key signaling pathway activation in response to PDGF-BB, including activation of ERK1/2, JNKs and P38, and PI3/kinase pathway. Together, these studies provide a thorough analysis of the differences in cell behavior that may explain the distinct biological functions of arterial and venous VSMCs.

## 2. Results

### 2.1. Differential Cell Proliferation, Migration and Dedifferentiation in Response to Growth Media (GM) or PDGF-BB

**Figure 1:**
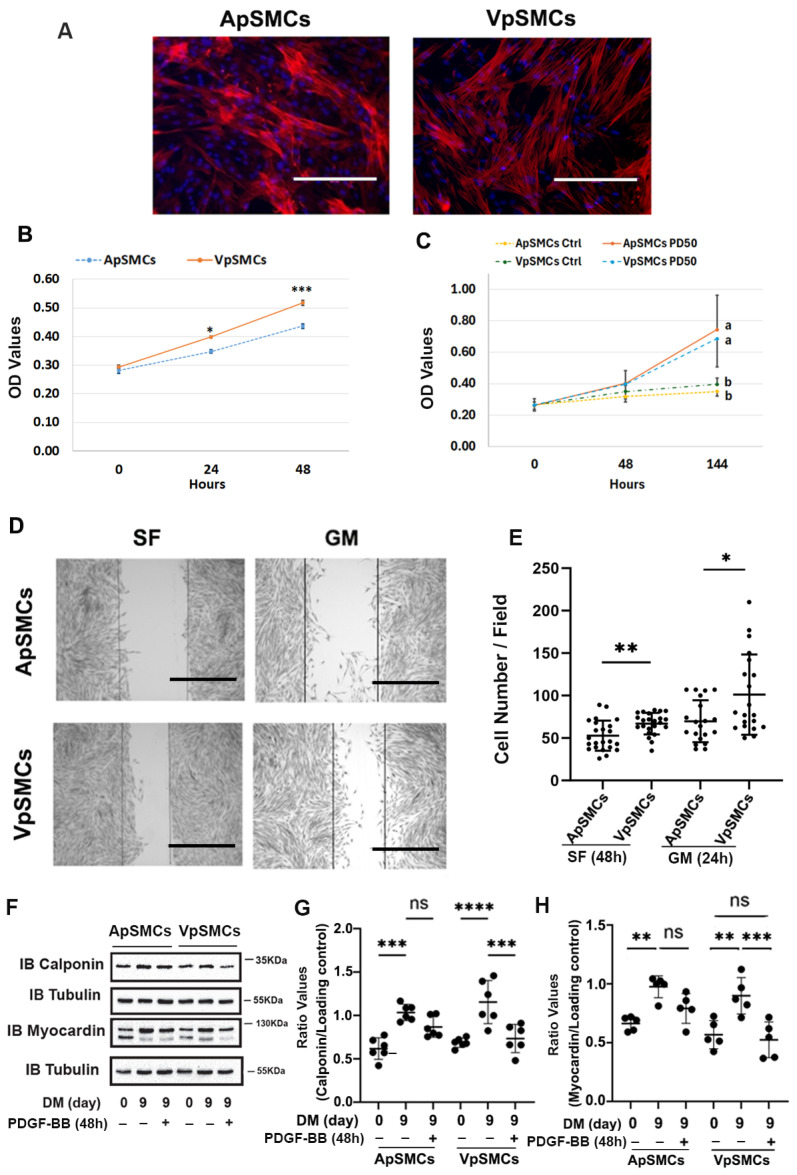
Differences in cell behavior between pig arterial smooth muscle cells (ApSMCs) and venous smooth muscle cells (VpSMCs). (**A**) The purity of VSMCs was demonstrated with anti-smooth muscle actin immunofluorescent staining, following the protocol described in the Materials and Methods. Scale bars = 200 microns. (**B**,**C**) Cell proliferation under the growth medium (GM) or in response to PDGF-BB stimulation was measured following the protocol described in the Materials and Methods. Different letters (a, b) indicated significant difference between two groups. (**D**) Cell migration assays were performed via scratch assays in serum-free (SF) or 10% FBS containing GM. (**E**) Migrated cells in scratched areas were counted. Scale bars = 200 microns. (**F**) Cells were induced to differentiate with a differentiation medium (DM), and 48 h before collecting cell lysates, PDGF-BB was applied in the indicated wells. (**G**,**H**) The densitometry of each band was obtained using Image J (NIH version 1.53K), and ratio values were obtained by interested protein bands/loading control bands. (*n* = 6 for calponin; *n* = 5 for myocardin.) ****: *p* < 0.000; ***: *p* < 0.001; **: *p* < 0.01; *: *p* < 0.05; ns: no significant differences were found using a Student’s *t*-test for two-group comparisons and an ANOVA test for multiple group comparisons.

To demonstrate the purity of isolated venous porcine VSMCs (VpSMCs) and arterial porcine VSMCs (ApSMCs), α-actin expression was examined in these cultured cells, which both showed similar levels of positive staining (Figure 1A). To examine cell proliferation, both the GM-stimulated and PDGF-BB-stimulated proliferation were determined. In response to GM, we observed significantly increased cell proliferation in VpSMCs compared to ApSMCs after 24 h (a 1.39 ± 0.13-fold vs. 1.25 ± 0.17-fold increase, *p* < 0.05) and 48 h (1.80 ± 0.21-fold vs. 1.56 ± 0.19-fold increase, *p* < 0.001) (Figure 1B). Although PDGF-BB-stimulated cell proliferation was robust in both subtypes of cells after 144 h of exposure, no differences between these two subtypes of cells was detected (Figure 1C). To evaluate potential cell migration differences, we performed cell scratch assays and observed enhanced cell migration for VpSMCs after 48 h under the serum-free condition (67 ± 12 vs. 53 ± 17 cells/field, *p* < 0.01) and 24 h under GM (101 ± 46 vs. 70 ± 24 cells/field, *p* < 0.05) compared to ApSMCs (Figure 1D,E). In addition, PDGF-BB-stimulated VSMCs’ phenotypic switch was investigated under differentiated conditions. After a 9-day exposure to differentiation media (DM), VSMCs’ differentiation markers, such as calponin and myocardin, were highly expressed. A 48-hour treatment with PDGF-BB (10 ng/mL) was able to induce significant cell dedifferentiation in VpSMCs—which was indicated by reducing differentiation marker protein expression—but not in ApSMCs, although there was a trend of reduction in these markers (Figure 1F–H). The data suggest that VpSMCs are more susceptible to pro-dedifferentiation environmental cues.

### 2.2. Cell Cycle-Related Proteins and ECM Proteins Expression

**Figure 2:**
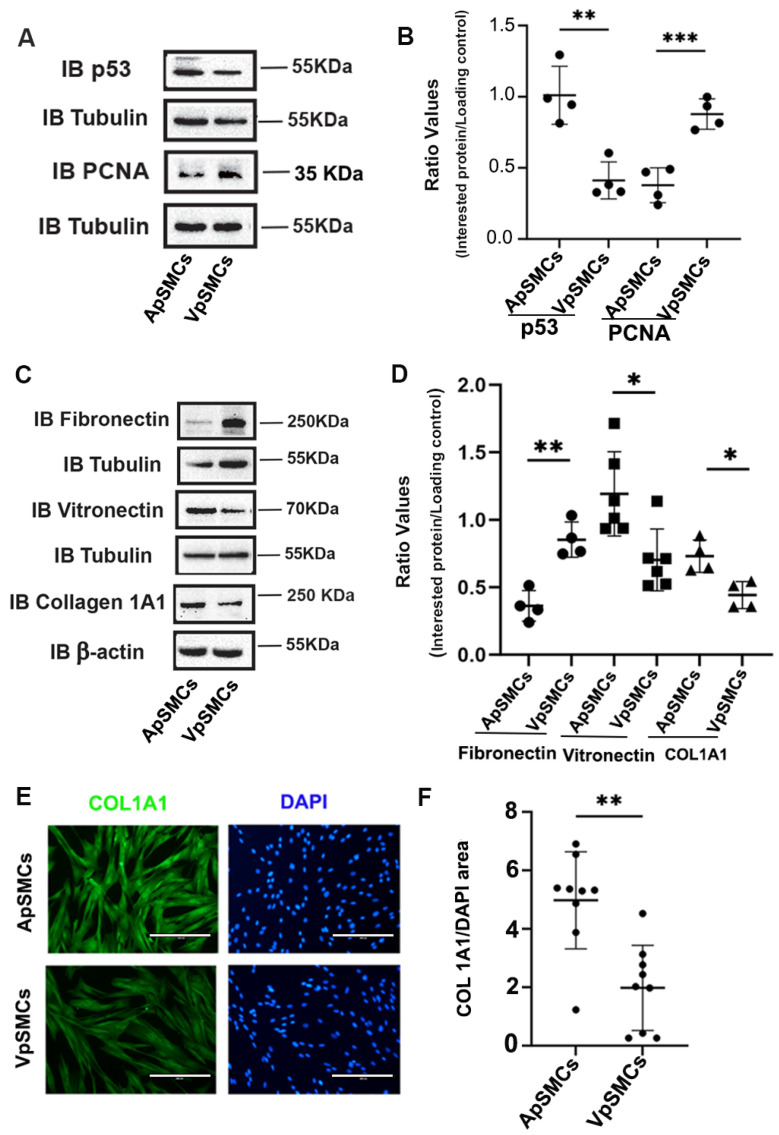
Differences in cell cycle proteins and extracellular matrix proteins between pig arterial smooth muscle cells (ApSMCs) and venous smooth muscle cells (VpSMCs). (**A**,**C**) Confluent cells were harvested and cell lysates were analyzed for the expression of the indicated proteins via immunoblots. (**B**,**D**) Individual band densitometry data were obtained using Image J (NIH version 1.53K). The ratio values were obtained by dividing the interested protein values by the loading protein values. (*n* = 4 for all parameters except vitronectin, which was *n* = 6.) (**E**) Cells were seeded in glass-bottom chambers and immunofluorescence staining was performed with an anti-collagen 1A1 (COL 1A1) antibody. Scale bars = 200 microns. (**F**) Fluorescence signals were quantified using Image J (NIH version 1.53K). ***: *p* < 0.001; **: *p* < 0.01; *: *p* < 0.05 were assessed using Student’s *t*-test.

The differences in cell proliferation drove us to investigate the expression of cell cycle-related proteins, including p53, which is a tumor suppressor protein that limits cell proliferation. Interestingly, the results indicated that the expression of the p53 protein was significantly lower in VpSMCs (by 60 ± 7% less, *p* < 0.01) compared to ApSMCs (Figure 2A,B). Consistent with our cell proliferation data, expression of the mitotic marker PCNA was higher in VpSMCs (increased 2.48 ± 0.66-fold, *p* < 0.001) compared to ApSMCs (Figure 2A,B).

Because ECM proteins significantly affect cell biological functions, we examined the expression profiles of several important ECM profiles in these two subtypes of VSMCs. Our results demonstrated that fibronectin expression was significantly increased in VpSMCs (a 2.46 ± 0.55-fold increase, *p* < 0.01), while vitronectin expression was significantly lower in VpSMCs (by 47 ± 16% less, *p* < 0.05) compared to ApSMCs (Figure 2C,D). In addition, collagen 1A1 (COL 1A1) expression was lower in VpSMCs (by 40 ± 6% less, *p* < 0.05) compared to ApSMCs (Figure 2C,D). This finding was confirmed by immunofluorescent (IF) staining for COL 1A1 (Figure 2E,F).

### 2.3. Focal Adhesion (FA) Proteins Expression

**Figure 3:**
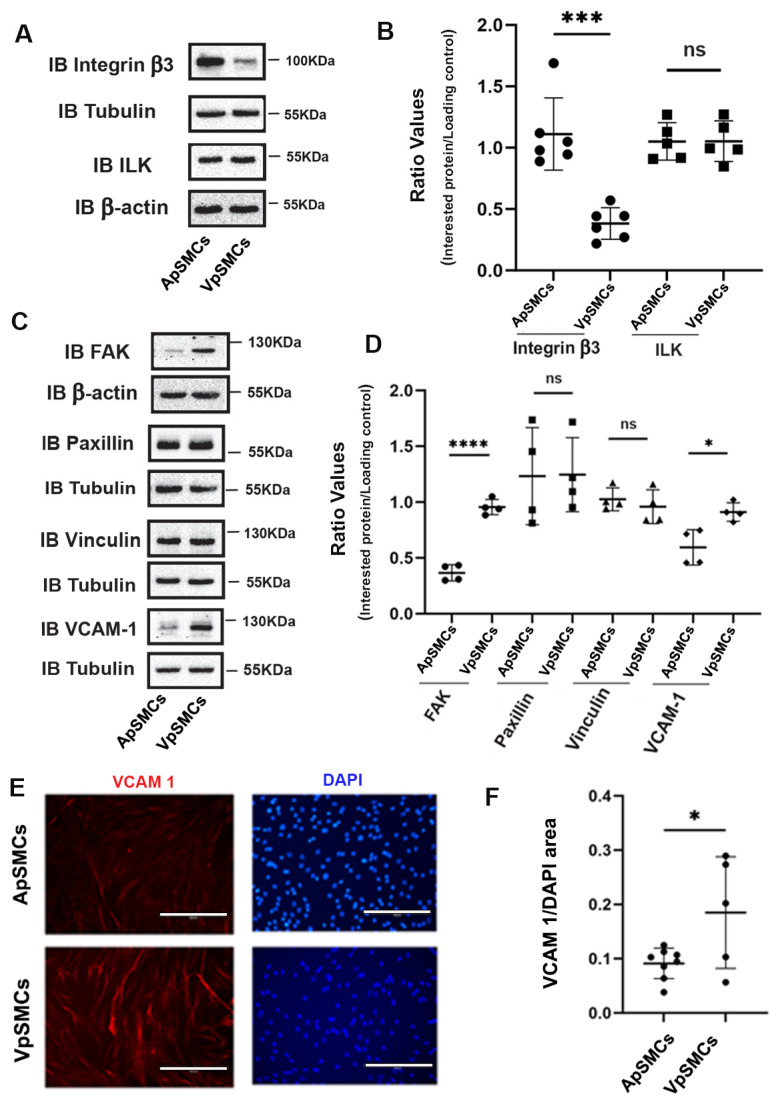
Differential expression of focal adhesive proteins between porcine arterial smooth muscle cells (ApSMCs) and venous smooth muscle cells (VpSMCs). (**A**,**C**) Confluent cells were harvested and cell lysates were analyzed for the expression of indicated proteins via immunoblots. (**B**,**D**) Individual band densitometry data were obtained using Image J (NIH version 1.53K). The ratio values were obtained by dividing the interested proteins values by the loading protein values (*n* = 6 for integrin β3; *n* = 5 for ILK; *n* = 4 for others). (**E**) Cells were seeded in glass-bottom chambers and immunofluorescent staining was performed with an anti-vascular cell adhesion molecule-1 (VCAM-1) antibody. Scale bars = 200 microns. (**F**) Fluorescent signals were quantified using Image J (NIH version 1.53K). ****: *p* < 0.0001; ***: *p* < 0.001; *: *p* < 0.05; ns: non-significance were all assessed using Student’s *t*-test.

Following our ECM proteins expression results, we were also interested in another group of proteins that are associated with ECM: FA proteins, which play critical roles in regulating cell anchoring and migration. Our initial focus was on the integrin β3 and integrin-linked kinase (ILK) proteins. Our results demonstrated that integrin β3 was expressed highly in ApSMCs (a 3.10 ± 0.93-fold increase, *p* < 0.0001) compared to VpSMCs, while the expression of ILK was similar across both subtypes of cells (Figure 3A,B). We also examined the expression of focal adhesion kinase (FAK), paxillin and vinculin in these cells. Our results showed that the FAK protein was highly expressed in VpSMCs (a 2.69 ± 0.58-fold increase over ApSMCs, *p* < 0.0001) but that paxillin and vinculin expression levels were similar (Figure 3C,D). Furthermore, vascular cell adhesion molecule-1 (VCAM-1) expression was higher in VpSMCs than ApSMCs, as determined by a Western blot and IF staining (Figure 3C–F).

### 2.4. MMPs and TIMPs Expressions

**Figure 4:**
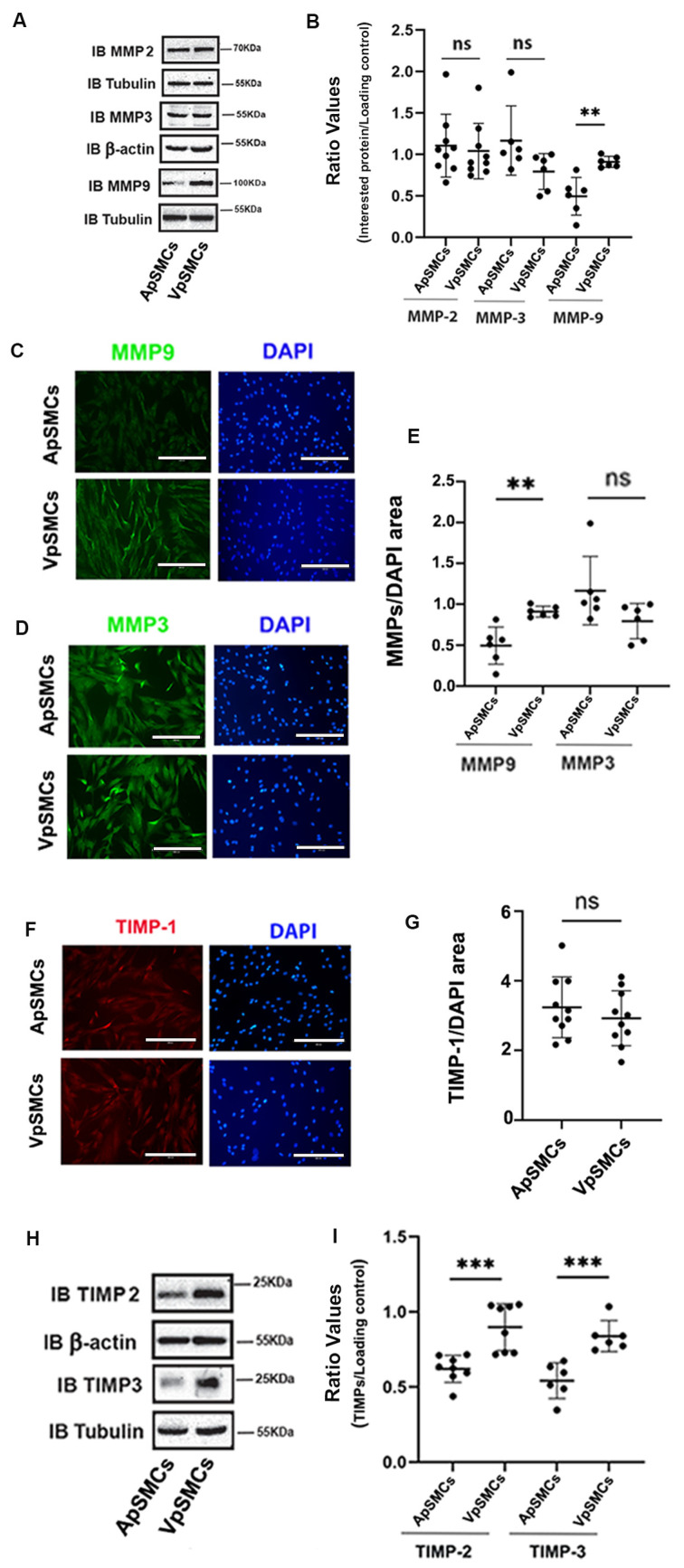
Differential expression of matrix metalloproteinases (MMPs) and tissue inhibitors of metalloproteinases (TIMPs) between pig arterial smooth muscle cells (ApSMCs) and venous smooth muscle cells (VpSMCs). (**A**,**H**) Confluent cells were harvested and cell lysates were analyzed for indicated protein expression via immunoblots. (**B**,**I**) Individual band densitometry data were obtained using Image J (NIH version 1.53K). The ratio values were obtained by dividing the interested protein values by the loading protein values (*n* = 9 for MMP-2; *n* = 6 for MMP-3, MMP-9 and TIMP-3; *n* = 8 for TIMP-2). (**C**,**D**,**F**) Cells were seeded in glass-bottom chambers and immunofluorescent staining was performed with anti-MMP-9 (**C**), anti-MMP-3 (**D**) or anti-TIMP-1 (**F**) antibodies. Scale bars = 200 microns. (**E**,**G**) Fluorescent signals were quantified using Image J (NIH version 1.53K) for MMP-9 and MMP-3 (**E**) and TIMP-1 (**G**). ***: *p* < 0.001; **: *p* < 0.01 and ns: non-significant differences were assessed using Student’s *t*-test.

In addition to ECM and FA proteins, the MMPs/TIMPs system also plays an important role in regulating VSMCs proliferation and migration. Therefore, MMP-2, MMP-3 and MMP-9 were examined. Our results indicated that MMP-9 was highly expressed in VpSMCs (1.73 ± 0.47-fold increase compared to ApSMCs, *p* < 0.01) while MMP-2 and MMP-3 expression levels were similar across the two subtypes of cells (Figure 4A,B). Meanwhile, IF results confirmed these findings for MMP-9 (Figure 4C,E) and MMP-3 (Figure 4D,E). Furthermore, the TIMPs expression data clearly demonstrated that TIMP-1 was similar in both subtypes of cells (Figure 4F,G), while TIMP-2 and TIMP-3 were more highly expressed in VpSMCs as compared to ApSMCs (Figure 4H,I).

### 2.5. Mitogen-Activated Protein (MAP) Kinases and AKT Activation in Response to PDGF-BB

**Figure 5:**
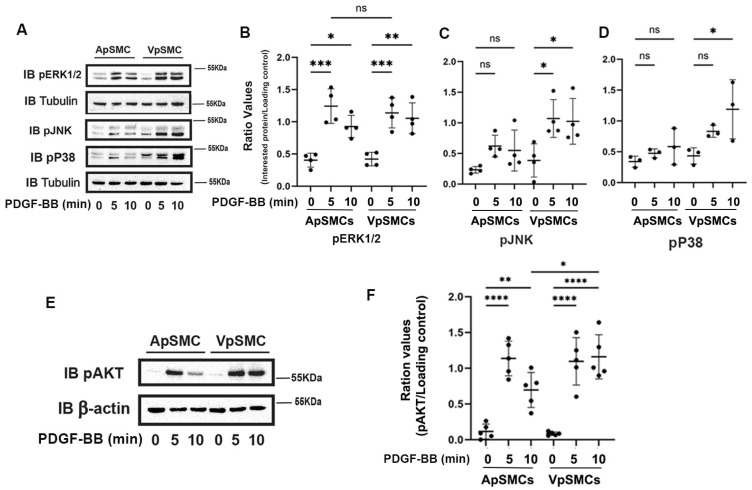
Differences in the activation of MAP kinases and AKT in response to PDGF-BB stimulation between pig arterial smooth muscle cells (ApSMCs) and venous smooth muscle cells (VpSMCs). (**A**,**E**) Confluent cells were serum-starved overnight and stimulated with PDGF-BB at the indicated time points. Cell lysates were analyzed for the expression of the indicated proteins via immunoblots. (**B**–**D**,**F**) Individual band densitometry data were obtained using Image J (NIH version 1.53K). The ratio values were obtained by dividing the band densitometry values of pERK1/2 (**B**), pJNK (**C**), pP38 (**D**) and pAKT (**F**) by the loading protein values (*n* = 4 for pERK1/2 and pJNK; *n* = 3 for pP38; *n* = 5 for pAKT). ****: *p* < 0.0001; ***: *p* < 0.001; **: *p* < 0.01; *: *p* < 0.05; ns: non-significant differences were assessed using an ANOVA test.

Since MAP kinases and AKT activation play crucial roles in mediating cell proliferation and migration, we investigated the activity of these two important pathways in response to PDGF-BB stimulation. In mammals, the MAP kinases can be grouped into three main families: extracellular-signal-regulated kinases (ERK1/2), Jun amino-terminal kinases (JNKs) and stress-activated protein kinases (P38/SAPKs). The activity of these pathways can be determined by quantifying the levels of phosphorylation of these key proteins (i.e., pERK1/2, pJNK and pP38). Our results demonstrated that after 5 and 10 min of PDGF-BB treatment, ERK1/2 signaling was activated equally in ApSMCs and VpSMCs (Figure 5A,B). In contrast, we observed that PDGF-BB barely stimulated the activation of JNKs and P38 in ApSMCs but significantly enhanced the phosphorylation of JNKs (after 5 and 10 min) and P38 (after 10 min) in VpSMCs (Figure 5A,C,D). In addition, phosphorylation of AKT after the PDGF-BB treatment showed a similar response in both subtypes of cells after a 5 min treatment, but there was a significantly stronger response in VpSMCs after 10 min of treatment as compared to ApSMCs (a 14.94 ± 5.73-fold vs. 5.54 ± 3.00-fold increase, *p* < 0.05) (Figure 5E,F). These results clearly indicate that different signaling pathways could be involved in VpSMCs’ and ApSMCs’ activation. Even when the same pathway was activated, the magnitude of activation in response to the same growth factor could differ significantly between the venous and arterial VSMCs.

## 3. Discussion

Venous neointimal hyperplasia and inward or negative remodeling following the creation of arteriovenous access have been considered as major contributors to arteriovenous fistulae and graft failures. Our current study revealed significant differences between venous and arterial VSMCs in several key aspects of relevant biological functions, including proliferation, migration and dedifferentiation. We further demonstrated numerous differences in the expression of cell cycle-related proteins, ECM proteins, FA proteins and MMPs/TIMPs, as well as differences in the activation of key MAP kinase and the AKT pathway. While these proteins and signaling pathways play crucial roles in regulating biological functions in both subtypes of VSMCs, the differences we identified in their expression/activation may help explain some of the key differences in the behavior and susceptibility to injury of these two subtypes of VSMCs.

To explore the underlying mechanisms that are responsible for venous and arterial VSMCs’ biological functional diversity, we first examined several important cell cycle-related proteins. Our data clearly demonstrated that p53, a tumor suppressor protein, was less expressed in VpSMCs than ApSMCs, which may contribute to enhanced venous VSMCs proliferation. In fact, this enhanced cell proliferation in VpSMCs was also observed in the regular maintenance of cell cultures. Interestingly, the expression of p53 has also been correlated with cell migration. A previous study showed that VSMCs isolated from p53-/- mice had significantly higher rates of migration and produced more invasive podosomes [8]. This observation was consistent with our current findings, which showed a lower expression of p53 in venous VSMCs and a higher cell migration ability compared to arterial VSMCs.

It is well known that ECM proteins significantly affect VSMCs’ biological functions, including proliferation, migration and vascular remodeling [9,10,11]. The current study determined the expression levels of fibronectin, vitronectin and COL1A1 in ApSMCs and VpSMCs, finding that fibronectin was highly expressed in VpSMCs but that vitronectin and COL1A1 were highly expressed in ApSMCs. These results suggest that the ECM composition and function of these two subtypes of VSMCs may differ significantly. An early study using a rat carotid artery injury model demonstrated that fibronectin is mainly expressed in proliferative and secretory VSMCs, while laminin and other basement membrane components promote the expression of differentiated VSMCs phenotypes [12], which supports the notion that different ECM proteins may be responsible for different physiological or pathophysiological functions.

In addition to ECM, FA proteins have also been shown to play critical roles in mediating cells’ biological functions, such as cell anchoring and migration in vitro and in vivo [13]. For example, integrin β3 has been previously reported to enhance VSMCs accumulation in neointimal hyperplasia after carotid ligation in mice [14]. Our current data revealed that integrin β3 was highly expressed in ApSMCs, which is consistent with its ligand, vitronectin, which was also highly expressed in ApSMCs compared to VpSMCs. In addition, our study revealed that FAK and VCAM-1 were expressed highly in VpSMCs compared to ApSMCs. FAK activation has been associated with enhanced neointimal hyperplasia in a wire injury femoral artery animal model [15]. In addition to regulating VSMCs proliferation, cytoplasm FAK activation has also been shown to stimulate VSMCs dedifferentiation via stabilizing DNA methyltransferase 3A, which maintains methylation of contractile genes while FAK nuclear translocation led to reduced methyltransferase 3A protein [16]. Meanwhile, a prior study has shown that VCAM-1 is required to stimulate VSMCs migration [17]. Therefore, increased expression of FAK and VCAM-1 may be, at least partially, contributing to the enhanced cell proliferation and migration that we observed in VpSMCs.

Since both MMPs and TIMPs play a vital role in regulating VSMCs migration, proliferation and apoptosis [18], we also examined the MMPs’ and TIMPs’ expression. A previous study showed that MMP-2 and MMP-9 expression was similar between the VSMCs isolated from a patient’s saphenous vein and internal mammary artery [4]. However, higher levels of MMP-2 and MMP-9, as well as TIMP-1 and -2, were detected in venous VSMCs isolated from the jugular veins of male white New Zealand rabbits [5]. Our comparison of VpSMCs and ApSMCs was more similar to the findings of this latter study, though we detected no difference in the expression of MMP-2 or TIMP-1. This discrepancy may represent variations in animal species or the different sources for the isolation of VSMCs.

PDGF-stimulated MAP kinase signaling, such as ERK1/2, plays a key role in mediating VSMCs proliferation and migration, as in other cell types [19,20]. Our data indicated that PDGF-BB was able to stimulate ERK1/2 activation in both ApSMCs and VpSMCs to a similar extent. In contrast, both PDGF-BB-stimulated JNKs and P38 activation were much more robust in VpSMCs compared to ApSMCs. PDGF-BB-stimulated JNK1 and P38 activation have been reported in rat aortic VSMCs [21] and in human arterial VSMCs, as well as saphenous vein VSMCs [19]. However, the non-activation of JNKs in response to PDGF-BB has also been reported in VSMCs [22] and porcine aortic endothelial cells [23]. In addition, one previous study demonstrated similar AKT activation in response to PDGF-BB in human arterial and venous VSMCs [19]. In this study, stronger AKT activation was detected in VpSMCs in response to PDGF-BB stimulation compared to ApSMCs. These discrepancies may be due to differences in the species or sources of VSMCs.

Although this study identified significant differences between arterial and venous VSMCs in the expression of numerous genes and in the activity of several signaling pathways, we recognize several limitations of the current study. We took a candidate gene approach to examining potential differences between VpSMCs and ApSMCs, based on known protein function and their potential significance to VSMCs behavior. Moving forward, we will also pursue a genome-wide comparison of VpSMCs and ApSMCs using RNA sequencing. Due to limitations in the methodologies used in this study, some cell biological functions related to the differences in gene expression and signaling pathway activation in these two subtypes of cells were unable to be tested. In future work, we plan to experimentally test the functional significance of the differences we described in gene expression and signaling pathways in these cells.

In summary, our current study reveals key differences in the behavior of porcine arterial VSMCs and venous VSMCs, including cell proliferation, migration and dedifferentiation susceptibility. In addition, we detected differential expression in many cellular functionality-related proteins, which may contribute to the differences we observed in the proliferation/cell cycle, focal adhesion, migration and dedifferentiation of these two subtypes of VSMCs. Furthermore, our study demonstrated the enhanced activation of P38, JNKs and AKT in venous VSMCs compared to arterial VSMCs (Table 1). We believe that these results provide vital information for the identification of specific molecules and pathways that can be preferentially activated in venous VSMCs as compared to arterial VSMCs. These molecules and pathways can potentially serve both as biomarkers for the identification of patients at greater risk of venous segment stenoses and, more importantly, as vein-specific therapeutic targets for those patients.

## 4. Materials and Methods

### 4.1. Materials

The Immobilon-P membrane, crystal violet blue (V5265), heparin sodium (H3149), anti-β-actin (A1978), anti-FAK (06-543), anti-vinculin (V9131), anti-MMP-2 (IM33) and anti-IRS-1 (05-1085) antibodies were purchased from MilliporeSigma (St. Louis, MO, USA). The anti-α-actin (SC-32251), anti-calponin (SC-136987), anti-MMP-9 (SC-21733) and anti-TIMP-1 (SC-365905) were obtained from Santa Cruz Biotechnology, Inc. (Dallas, TX, USA). The anti-α-tubulin (GTX112141) antibody was purchased from GeneTex (Irvine, CA, USA). The PDGF-BB (220-BB) and anti-myocardin (MAB4028) antibody were purchased from R&D Systems (Minneapolis, MN, USA). The anti-p53 (10442-1-AP), anti-PCNA (10205-2-AP), anti-fibronectin (15613-1-AP), anti-vitronectin (15833-1-AP) and anti-MMP-3 (66338-1-lg) antibodies were purchased from Proteintech (Rosemont, IL, USA). The anti-collagen 1A1 (A22090), anti-VCAM-1 (A23692) and KLF-4 (A13673) were obtained from ABclonal (Woburn, MA, USA). Another anti-collagen 1A1 (600-401-1103) for the cell immunofluorescence study was purchased from Rockland (Limerick, PA, USA). The anti-integrin β3 (ab19992) and anti-paxillin (ab32084) antibodies were purchased from Abcam (Cambridge, UK). The anti-ILK (3862), anti-TIMP-2 (5738), anti-pERK1/2(9101), anti-pJNK (9251), anti-pP38 (9211) and anti-pAKT (Serine 473) (9271) antibodies were purchased from Cell Signaling Technology, Inc. (Danvers, MA, USA). The anti-TIMP-3 antibody (Orb214673) was purchased from Biorbyt (Durham, NC, USA). MTT (Cat# 21795) was purchased from Cayman Chemical (Ann Arbor, MI, USA). All other reagents were obtained from MilliporeSigma unless otherwise stated.

### 4.2. Cell Culture

ApSMCs and VpSMCs were isolated from the carotid artery and jugular vein, respectively, of a 4-month-old Yorkshire pig. Cells were cultured with GM (DMEM containing 5 mM of glucose and 10% FBS plus 1% penicillin/streptomycin (P/S)). All experiments were performed on cells between passage 4 and passage 10. For the differentiation studies, the confluent cells were exposed to DM (DMEM containing 1% FBS, 1% P/S and 30 ug/mL of heparin) for the indicated time spans. For growth factor treatment, 10 ng/mL of PDGF-BB was applied to the medium for the indicated time span. Cell lysates were collected to analyze the protein expression levels.

### 4.3. Cell Proliferation Assay

Cell proliferation was measured by an MTT assay. Briefly, 2000 cells/well were seeded into a 96-well plate. Cells were cultured in the GM or starving media (0.5% FBS containing DMEM). For the GM experiment, MTT was applied to the well after 24 or 48 h of exposure to GM. For the growth factor treatment, PDGF-BB (50 ng/mL) was applied every 48 h. After 48 h or 144 h of exposure to PDGF-BB, 10 uL of MTT (12 mM) was added to each well. After 4 h of incubation, 0.01 N of HCl with a 10% SDS solution was added to each well and incubated overnight before measuring with a microplate reader for OD570.

### 4.4. Cell Migration Assay

The cell migration assay was measured by scratch assays. Cells (1.5 × 10^5^) were seeded on a 6-well plate and exposed to GM until confluency. Cells were scratched by a pipette tip. The basal scratch areas were imaged by an EVOS microscope (ThermoFisher Scientific, Waltham, MA, USA). After 24 h, for GM, or 48 h, for PDGF-BB, of treatment in the serum-free medium, cells were fixed with 10% formalin and stained with crystal violet blue before being imaged by the EVOS system. Cells that crossed the scratch lines were considered to be migrating cells.

### 4.5. Immunoblotting

The cell monolayers were lysed in a modified radioimmunoprecipitation assay (RIPA) buffer, as previously described [24]. Immunoblotting was performed, as previously described [24], using a dilution of 1:3000 for the anti-calponin, anti-PCNA, anti-tubulin, anti-pERK1/2 and anti-β-actin antibodies; a dilution of 1:1000 for the anti-P53, anti-fibronectin, anti-vitronectin, anti-collagen 1A1, anti-integrin β3, anti-ILK, anti-FAK, anti-paxillin, anti-vinculin, anti-VCAM-1, anti-MMP-9, anti-MMP-3, anti-MMP-2, anti-TIMP-1, anti-TIMP-2, anti-TIMP-3 and anti-pAKT antibodies; and a dilution of 1:500 for the anti-myocardin, anti-IRS-1, anti-pJNK and anti-pP38 antibodies. The proteins were visualized using enhanced chemiluminescence (Thermo Fisher Scientific, Rockford, IL, USA). The total cellular protein in the lysates was determined using a bicinchoninic acid assay (Thermo Fisher Scientific, Rockford, IL, USA).

### 4.6. Immunofluorescent Staining

For the IF staining experiments, the cells were seeded in a µ-Slide 4-well plate (Ibidi, Cat# 80426), and grown until 80% confluency. The cells were fixed with 4% paraformaldehyde, permeabilized with 0.2% Triton-X 100 and then incubated with anti-α-actin (1:200), anti-collagen 1A1 (1:200), anti-VCAM-1 (1:250), anti-MMP-9 (1:250), anti-MMP-3 (1:250) or anti-TIMP-1 (1:200) antibodies overnight in a cold room. Cells were covered with a mounting medium containing DAPI (H-1200, Vector laboratories, Inc., Newark, CA, USA). Images were captured by an EVOS fluorescence microscope (ThermoFisher Scientific). The IF quantification data were obtained, following the protocol described previously [25], by measuring the area of specific-staining images divided by the area of DAPI-staining images using Image J (NIH version 1.53K).

### 4.7. Statistical Analysis

Densitometry results are expressed as the mean ± standard deviation (SD). All experiments were replicated at least three times to ensure reproducibility. The Western blot images were captured by an iBright 1000 (ThermoFisher Scientific). The densitometry was obtained by Image J (NIH version1.53K). The results were analyzed for statistically significant differences using Student’s *t*-test when two groups were compared, or ANOVA was applied when multiple groups were compared. All statistical analyses and figures were generated using GraphPad Prism (version 10.1.0 for Windows, GraphPad Software, Boston, MA, USA). Statistical significance was set at *p* < 0.05.

## Figures and Tables

**Table 1:** Differences in biological function, protein expression and signal pathway activation in venous and arterial vascular smooth muscle cells (VSMCs).

Cell Subtypes	Biological Functions ^1^	Cell Cycle-Related Proteins ^2^	ECM Proteins ^2^	Focal Adhesion Proteins ^2^	MMPs/TIMPs ^2^	MAP Kinase and PI3/Kinase ^1^
Venous VSMCs	Proliferation (↑↑) Migration (↑↑) Dedifferentiation (↑↑)	P53 (↓) PCNA (↑)	Fibronectin (↑) Vitronectin (↓) Collagen 1A1 (↓)	Integrin β3 (↓) ILK (↔) FAK (↑) Paxillin (↔) Vinculin (↔) VCAM-1 (↑)	MMP-2 (↔) MMP-3 (↔) MMP-9 (↑) TIMP-1 (↔) TIMP-2 (↑) TIMP-3 (↑)	pERK1/2(↑) pJNKs (↑↑) pP38 (↑↑) pAKT (↑↑)
Arterial VSMCs	Proliferation (↑) Migration (↑) Dedifferentiation (↑ or ↔)	P53 (↑) PCNA (↓)	Fibronectin (↓) Vitronectin (↑) Collagen 1A1 (↑)	Integrin β3 (↑) ILK (↔) FAK (↓) Paxillin (↔) Vinculin (↔) VCAM-1 (↓)	MMP-2 (↔) MMP-3 (↔) MMP-9 (↓) TIMP-1 (↔) TIMP-2 (↓) TIMP-3 (↓)	pERK1/2 (↑) pJNKs (↑ or ↔) pP38 (↑ or ↔) pAKT (↑)

^1^ ↑ denotes an increase in response to the growth medium or PDGF-BB; ↔ denotes no response to PDGF-BB stimulation; ^2^ ↑ denotes higher expression; ↓ denotes lower expression; ↔ denotes no difference in the expression of the indicated proteins between venous VSMCs and arterial VSMCs.

## Data Availability

The authors declare that all data supporting the findings of this study will be available from the corresponding authors upon request.
